# Lack of association between screening interval and cancer stage in Lynch syndrome may be accounted for by over-diagnosis; a prospective Lynch syndrome database report

**DOI:** 10.1186/s13053-019-0106-8

**Published:** 2019-02-28

**Authors:** Toni T. Seppälä, Aysel Ahadova, Mev Dominguez-Valentin, Finlay Macrae, D. Gareth Evans, Christina Therkildsen, Julian Sampson, Rodney Scott, John Burn, Gabriela Möslein, Inge Bernstein, Elke Holinski-Feder, Kirsi Pylvänäinen, Laura Renkonen-Sinisalo, Anna Lepistö, Charlotte Kvist Lautrup, Annika Lindblom, John-Paul Plazzer, Ingrid Winship, Douglas Tjandra, Lior H. Katz, Stefan Aretz, Robert Hüneburg, Stefanie Holzapfel, Karl Heinimann, Adriana Della Valle, Florencia Neffa, Nathan Gluck, Wouter H. de Vos tot Nederveen Cappel, Hans Vasen, Monika Morak, Verena Steinke-Lange, Christoph Engel, Nils Rahner, Wolff Schmiegel, Deepak Vangala, Huw Thomas, Kate Green, Fiona Lalloo, Emma J. Crosbie, James Hill, Gabriel Capella, Marta Pineda, Matilde Navarro, Ignacio Blanco, Sanne ten Broeke, Maartje Nielsen, Ken Ljungmann, Sigve Nakken, Noralane Lindor, Ian Frayling, Eivind Hovig, Lone Sunde, Matthias Kloor, Jukka-Pekka Mecklin, Mette Kalager, Pål Møller

**Affiliations:** 1 Department of Surgery, Helsinki University Central Hospital, P.O. Box 340, 00029 HUS Helsinki, Finland; 2 University of Helsinki, Helsinki, Finland; 3 Heidelberg University Hospital and DKFZ, Heidelberg, Germany; 4 Department of Tumor Biology, Institute of Cancer Research, The Norwegian Radium Hospital, part of Oslo University Hospital, Olso, Norway; 5 Department of Medical Genetics, The Norwegian Radium Hospital, Oslo University Hospital, Oslo, Norway; 6 The Royal Melbourne Hospital, Melbourne, Australia; 7 University of Melbourne, Melbourne, Australia; 8 University of Manchester & Manchester University Hospitals Foundation Trust, Manchester, UK; 9 The Danish HNPCC Register, Clinical Research Centre, Copenhagen University Hospital, Hvidovre, Denmark; 10 Medical Genetics, Cardiff University, Cardiff, UK; 11 University of Newcastle and the Hunter Medical Research Institute, Callaghan, Australia; 12 University of Newcastle, Newcastle upon Tyne, UK; 13 University Witten-Herdecke, Wuppertal, Germany; 14 Dept. of Surgical Gastroenterology, Aalborg University Hospital, Aalborg, Denmark; 15 Medizinische Klinik und Poliklinik IV, Campus Innenstadt, Klinikum der Universität München, Munich, Germany; 16 MGZ- Medical Genetics Center, Munich, Germany; 17 Central Finland Central Hospital, Education and Research, Jyväskylä, Finland; 18 Department of Clinical Genetics, Aalborg University Hospital, Aalborg, Denmark; 19 Karolinska Institutet, Stockholm, Sweden; 20 Hadassah Medical Center, Jerusalem, and Sheba Medical Center, Tel-Hashomer, Ramat-Gan, Israel; 21 Institute of Human Genetics, University of Bonn, Bonn, Germany; 22 Department of Internal Medicine I, University Hospital Bonn, Bonn, Germany; 23 Center for Hereditary Tumor Syndromes, University Hospital Bonn, Bonn, Germany; 24 Institute for Medical Genetics and Pathology, University Hospital Basel, Basel, Switzerland; 25 Hospital Fuerzas Armadas, Grupo Colaborativo Uruguayo, Investigación de Afecciones Oncológicas Hereditarias (GCU), Montevideo, Uruguay; 26 Tel-Aviv Soursky Medical Center, Tel-Aviv, Israel; 27 Department of Gastroenterology and Hepatology, Isala Clinics, Zwolle, The Netherlands; 28 Department of Gastroenterology and Hepatology, Leiden University Medical Centre, Leiden, The Netherlands; 29 Institute for Medical Informatics, Statistics and Epidemiology, University of Leipzig, Leipzig, Germany; 30 Medical School, Institute of Human Genetics, Heinrich-Heine-University, Düsseldorf, Germany; 31 Department of Medicine, Knappschaftskrankenhaus, Ruhr-University Bochum, Bochum, Germany; 32 St Mark’s Hospital, Department of Surgery and Cancer, Imperial College London, London, UK; 33 University of Manchester and St Mary’s Hospital, Manchester, UK; 34 Hereditary Cancer Program, Catalan Institute of Oncology, Insititut d’Investigació Biomèdica de Bellvitge (IDIBELL), ONCOBELL Program, L’Hospitalet de Llobregat, Barcelona, Spain; 35 Centro de Investigación Biomédica en Red de Cáncer (CIBERONC), Barcelona, Spain; 36 University Medical Center Groningen, Groningen, the Netherlands; 37 Leids Universitair Medisch Centrum, Leiden, Netherlands; 38 Department of Surgical Gastroenterology, Aarhus University Hospital, Aarhus, Denmark; 39 Department of Health Sciences Research, Mayo Clinic, Scottsdale, AZ USA; 40 Center for Bioinformatics, Department of Informatics, University of Oslo, Oslo, Norway; 41 Department of Medical Genetics, Aarhus University Hospital, Aarhus, Denmark; 42 Department of Surgery, Central Finland Central Hospital, Jyväskylä, Finland; 43 Faculty of Sport and Health Sciences, University of Jyväskylä, Jyväskylä, Finland; 44 University of Oslo, Oslo, Norway; 45 Harvard School of Public Health, Boston, MA USA

**Keywords:** Mismatch repair, Microsatellite instability, Lynch syndrome, Hereditary cancer, Colorectal cancer, Hereditary nonpolyposis colorectal cancer, Colonoscopy, Endoscopy, Surveillance, Screening, Over-diagnosis

## Abstract

**Background:**

Recent epidemiological evidence shows that colorectal cancer (CRC) continues to occur in carriers of pathogenic mismatch repair (*path_MMR*) variants despite frequent colonoscopy surveillance in expert centres. This observation conflicts with the paradigm that removal of all visible polyps should prevent the vast majority of CRC in *path_MMR* carriers, provided the screening interval is sufficiently short and colonoscopic practice is optimal.

**Methods:**

To inform the debate, we examined, in the Prospective Lynch Syndrome Database (PLSD), whether the time since last colonoscopy was associated with the pathological stage at which CRC was diagnosed during prospective surveillance. *Path_MMR* carriers were recruited for prospective surveillance by colonoscopy. Only variants scored by the InSiGHT Variant Interpretation Committee as class 4 and 5 (clinically actionable) were included. CRCs detected at the first planned colonoscopy, or within one year of this, were excluded as prevalent cancers.

**Results:**

Stage at diagnosis and interval between last prospective surveillance colonoscopy and diagnosis were available for 209 patients with 218 CRCs, including 162 *path_MLH1*, 45 *path*_*MSH2*, 10 *path_MSH6* and 1 *path_PMS2* carriers. The numbers of cancers detected within < 1.5, 1.5–2.5, 2.5–3.5 and at > 3.5 years since last colonoscopy were 36, 93, 56 and 33, respectively. Among these, 16.7, 19.4, 9.9 and 15.1% were stage III–IV, respectively (*p* = 0.34). The cancers detected more than 2.5 years after the last colonoscopy were not more advanced than those diagnosed earlier (*p* = 0.14).

**Conclusions:**

The CRC stage and interval since last colonoscopy were not correlated, which is in conflict with the accelerated adenoma-carcinoma paradigm. We have previously reported that more frequent colonoscopy is not associated with lower incidence of CRC in *path_MMR* carriers as was expected. In contrast, point estimates showed a higher incidence with shorter intervals between examinations, a situation that may parallel to over-diagnosis in breast cancer screening. Our findings raise the possibility that some CRCs in *path_MMR* carriers may spontaneously disappear: the host immune response may not only remove CRC precursor lesions in *path_MMR* carriers, but may remove infiltrating cancers as well. If confirmed, our suggested interpretation will have a bearing on surveillance policy for *path_MMR* carriers.

## Background

It is commonly agreed that adenomas in the colon may develop to infiltrating cancers, but the probability for one single adenoma to do so within a few years is low [1]. Most hereditary colorectal cancers (CRCs) are not associated large numbers of adenomas in the intestine, and to indicate that this is different from the situation in familial adenomatous polyposis, the term “hereditary non-polyposis colorectal cancer” (HNPCC) was agreed [2]. HNPCC patients often had a few adenomas in the large bowel at the time of CRC diagnosis, and Jass and Stewart proposed that *“adenomas do not occur in large numbers in HNPCC, but develop at a young age, attain a larger size, often show a villous configuration, and are more prone to malignant conversion than sporadic adenomas*”. This was referred to as the *“accelerated adenoma-carcinoma sequence”* and became accepted as the cause of CRC in HNPCC [3]. Consequently, it was assumed that colonoscopy with removal of macroscopically visible adenomas would prevent CRC in individuals at risk for HNPCC. It was soon documented that colonoscopy with a three-year interval reduced both CRC incidence and mortality in HNPCC kindreds [4, 5]. Compared with non-HNPCC moderate-risk familial CRC, even single colonoscopy at middle-age substantially reduced risk for subsequent CRC [6, 7] and there was no benefit from 3-yearly colonoscopies over 6-yearly colonoscopies [8].

However, CRC continued to occur despite intensive surveillance [9]. In an attempt to reduce incident CRCs, Vasen and others proposed shortening the interval between colonoscopies from 2 to 3 years to 1–2 years [10], now adopted as an international clinical guideline [11]. It was also agreed that extended surgery could be considered in individuals with HNPCC and CRC, recognizing that secondary prevention of CRC by colonoscopy surveillance was not always reliable [11].

The mismatch repair (MMR) mechanism is ubiquitous in nature as a means of repairing DNA damage [12, 13], and pathogenic variants in four genes: *MLH1, MSH2, MSH6* and *PMS2* were identified in HNPCC [14–17]*.* When cells with inherited pathogenic variants acquire a second somatic mutation that inactivates the wild-type allele, the consequence may be an MMR protein deficient tumor with microsatellite instability (MSI). In view of the wide extracolonic cancer phenotype, HNPCC was re-named Lynch syndrome (LS) and restricted to inherited cancer caused by a *path_MMR* gene variant [18].

It has long been suspected that some cancers do not develop through adenomas in LS, but rather through a pathway without a visible precursor polyp. The paradigm that all CRC in LS could be prevented by removal of macroscopically visible precursor lesions was not confirmed, even with more frequent surveillance colonoscopy [9, 10]. The Prospective Lynch Syndrome Database (PLSD) was established to prospectively follow *path_MMR* carriers. A key question was to validate the paradigm that removal of macroscopically visible adenomas during repetitive colonoscopies would prevent CRC*.* We have previously reported that the observed lifetime cumulative incidence of CRC in *path_MLH1* and *path_MSH2* carriers was 43–45% despite follow-up according to national and international guidelines [19–22].

To further inform the epidemiology of CRC in *path_MMR* carriers subjected to repeated colonoscopies, we examined the association between the time since last colonoscopy and pathological stage of prospectively detected CRC.

## Material and methods

The PLSD database design and its inclusion criteria have been described in detail previously [19, 21]. PLSD is an international, multicentre database recording prospective observational data on *path_MMR* carriers under surveillance by colonoscopy. All *path_MMR* carriers in each participating centre were included in the study. The demonstrated genetic variants were assumed inherited and were found by genetic testing either prior to, at, or after inclusion for follow-up. Only variants scored by the InSiGHT Governance Committee as class 4 and 5 (clinically actionable) were included. All cancers detected prior to, at or within one year after the age at the first planned and performed colonoscopy, were scored as prior or prevalent cancers and excluded from the analysis when scoring prospectively observed cancers. The surveillance strategies recommended by each participating centre are presented in Table 1.

**Table 1:** Clinical follow-up as carried out by each contributing centre

Centre	Series censored	Colonoscopy		Gynecological examination			References for details
		Interval	Time period	Interval	From-to	Modalities in addition to clinical examination	
Norway	2013	3 years	1989–1996	2 years	1989–2013	TV US CA12–5	[35]
		2 years (1 year when adenoma)	1996–2013				
Finland	2014	3 years	1985–2014	1 year	1995–2014	TV US Endometrial biopsy, CA12–5	[5, 36, 37]
Sweden	2014	2 years	1990–2000	1 year	1992–2014	TV US In most: CA 12–5 In some: endometrial biopsy	[38, 39]
		18 months	2000–2014				
Denmark	2014	2 years	1991–2014	2 years	1991–2014	TV US	[40]
The Netherlands	2013	2–3 years	1987–1996	1–2 years	1994–2005	TV US	[10]
		2 years	1996–2013	1–2 years	2005–2013	TV US endometrial biopsy	
Newcastle UK	2014	2 years	1995–2014	No fixed policy			
Manchester UK	2014	2 years	1994–2014	1 year	1990–2014	Hysteroscopy Ovarian US Ca12–5	[41]
Spain	2013	1–2 years (1 year when age > 40 years)	1999–2013	1 year	1999–2013	TV US	
Melbourne Australia	2014	Annual	1990–2014	Annual	1990–2005	TV US Endometrial biopsy CA12–5	https://wiki.cancer.org.au/australiawiki/index.php?oldid=86684 https://wiki.cancer.org.au/australiawiki/index.php?oldid=175314
					2005-	Risk reducing surgery only	

*TV US* transvaginal ultrasound

The following information was used in the statistical analyses: sex, *path_MMR* variant, age at inclusion, age at last update, age at CRC, type of cancer as indicated by the first three positions in the International Classification of Diseases version 9 (ICD-9) diagnostic system, the American Joint Committee on Cancer (AJCC) stage of CRC (I–IV) and the time since the last colonoscopy preceding the diagnosis of CRC. All cancers, including cancers prior to or at inclusion, were recorded for each carrier. Inclusion was from the first prospectively planned and completed colonoscopy, and all recruits had subsequent follow-up of one year or more.

Patients with a CRC detected during prospective follow-up were studied in detail. The time since the colonoscopy before the one in which the cancer diagnosis was established, was recorded in months and categorized as < 1.5, 1.5 to 2.5, 2.5 to 3.5 or > 3.5 years since last colonoscopy. We considered stage III-IV as advanced. We compared stage in the different time intervals since last colonoscopy, corresponding with the different clinical guidelines advocating 1, 2 or 3 year intervals between colonoscopies, as slightly longer intervals may occur for several reasons in a clinical setting. We also compared stage in dichotomized time intervals, more and less than 2.5 years, to compare the most common current recommendation of 2 years to longer intervals. We performed a sensitivity analysis excluding Finland since patients there were recommended 2–3-yearly colonoscopies rather than 1–2-yearly as in the other countries and because one out of two variants in the Finnish series was not shared by others. Statistical testing was performed by SPSS version 23 (IBM, Armonk, NY, US). Chi-square test was used to test the statistical significance at the level of *α* = 0.05 between the different colonoscopy intervals.

All collaborating centres undertook genetic testing according to national policies. No individually identifiable data was exported to the PLSD.

## Results

In the most recently updated series of the PLSD, 6350 pathogenic variant carriers of mismatch repair genes (*path_MMR)* were prospectively observed for 51,646 follow-up years. A total of 707 CRCs were detected, and stage and interval between last prospective surveillance colonoscopy and diagnosis were available for 209 patients with 218 (30.8%) CRCs (9 patients had a metachronous cancer during follow-up). There were 162 *path_MLH1*, 45 *MSH2*, 10 *path_MSH6* and 1 *path_PMS2* carriers.

**Fig. 1:**
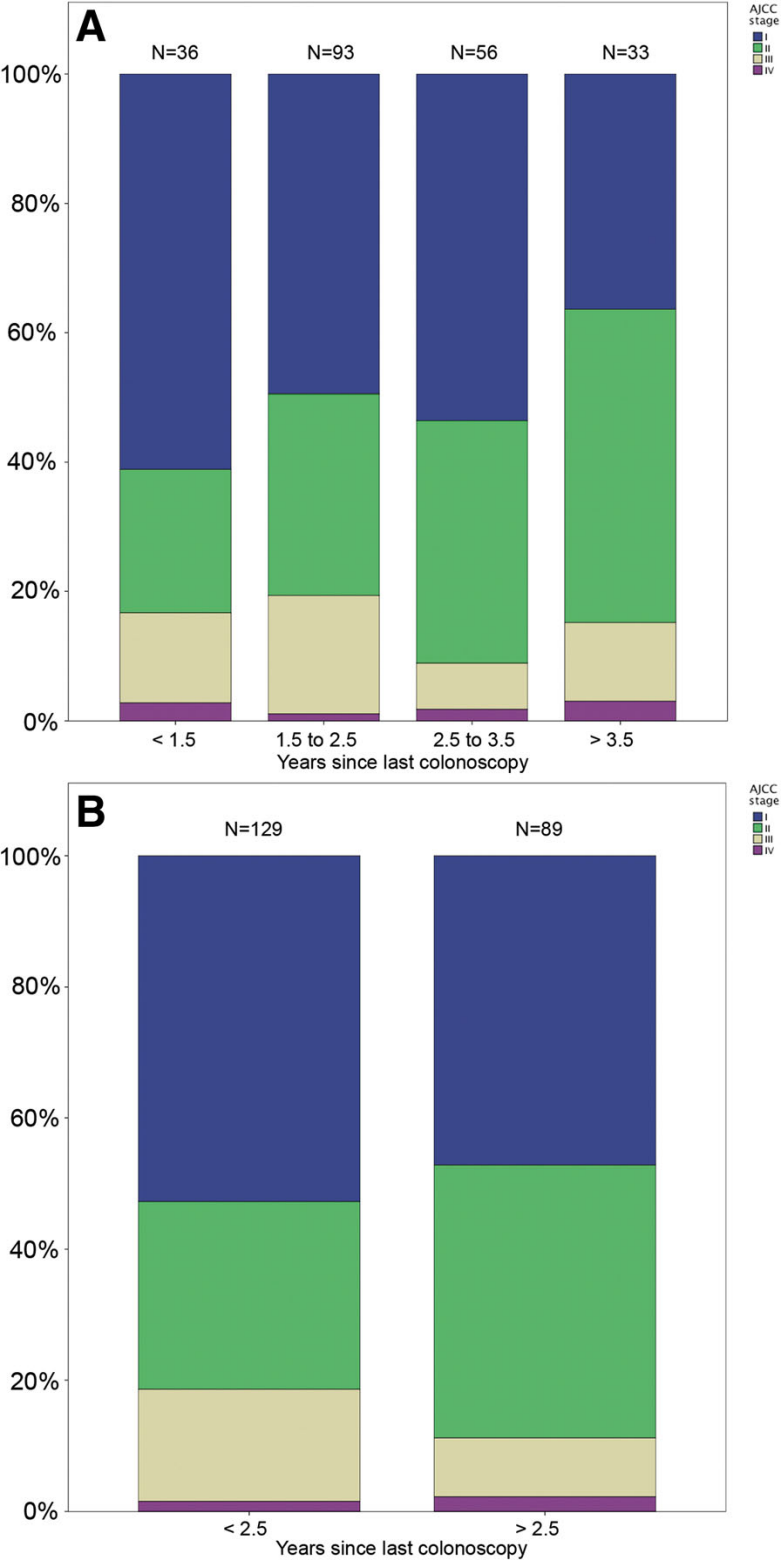
Number of CRC in different AJCC stages diagnosed in the time interval since the last surveillance colonoscopy. **a** time since last colonoscopy in intervals of < 1.5, 1.5 to 2.5, 2.5 to 3.5 and > 3.5 years. **b** Time intervals of less and more than 2.5 years

The numbers of cancers detected within < 1.5, 1.5 to 2.5, 2.5 to 3.5 and > 3.5 years since last colonoscopy were 36, 93, 56 and 33. Of these CRCs, 16.7, 19.4, 9.9 and 15.1% were advanced stage (III–IV), respectively (*p* = 0.40; Fig. 1a). The distribution of cancer stage were similar in the different colonoscopy intervals, *p* = 0.34 (Table 2). The cancers detected more than 2.5 years after the last colonoscopy were not more advanced than those diagnosed earlier than 2.5 years (*p* = 0.14; Fig. 1b).

**Table 2:** Stage distribution by the time since last colonoscopy before cancer diagnosis

	Less than 1.5 years (%)	1.5 to 2.5 years (%)	2.5 to 3.5 years (%)	Over 3.5 years (%)
Stage I	22 (61.1)	46 (49.5)	30 (53.6)	12 (36.4)
Stage II	8 (22.2)	29 (31.2)	21 (37.5)	16 (48.5)
Stage III	5 (13.9)	17 (18.3)	4 (7.1)	4 (12.1)
Stage IV	1 (2.8)	1 (1.1)	1 (1.8)	1 (1.8)
All stages	36 (100)	93 (100)	56 (100)	33 (100)

When excluding patients from Finland, we found a similar pattern as when analyzing the whole cohort. A total of 105 patients were included and 110 cancers were detected at follow-up. The numbers of cancers detected within < 1.5, 1.5 to 2.5, 2.5 to 3.5 and ≥ 3.5 years since last colonoscopy were 22 (20%), 53 (48.2%), 15 (13.6%) and 20 (18.2%). Of these CRCs, 18.2, 26.4, 6.7 and 15.0% were advanced stage (III–IV), respectively (*p* = 0.34).

Mean age at cancer diagnosis was 55 years. There was no difference between the colonoscopy interval distribution of those under (*n* = 110) and those over (*n* = 108) 55 years (*p* = 0.138). Stage III cancers were slightly more frequent among those under 55 years than those over 55 years (22 versus 8, respectively; *p* = 0.045).

## Discussion

We found that CRC stage distribution was not dependent on time since last colonoscopy. Stage distribution was similar irrespective of time since the previous colonoscopy, indicating that additional carcinogenetic mechanisms besides the accelerated adenoma-carcinoma pathway may have been instrumental in the development of some CRCs. Our findings are supported by another prospective study based on 16,327 colonoscopies in 2747 patients with *path_MMR* carriers [23], which found no correlation between advanced stage CRC and time since last colonoscopy.

There is convincing evidence that regular colonoscopy surveillance reduces CRC incidence and mortality in *path_MMR* carriers compared to no surveillance [4, 5], although the surveillance does not prevent the cancers as much as might had been expected [19–22]. The additional benefit of surveillance strategies with shorter interval remains less clear, since the study by Engel et al. showed no difference of CRC incidence between 1-, 2- or 3-yearly strategies [23]. We have previously shown that the recommended interval of 3 years lead to lower point estimate of cumulative incidence of CRC compared to recommended 1–2 year intervals [22], which was in conflict with the expected outcome of shorter than 3 years interval [10]. The purpose of the current analysis was to study if shorter time between colonoscopies would result in less advanced cancer stage when CRC was diagnosed. Although this was not a direct comparison of different surveillance strategies, reduced stage was not found to be the additional benefit of more frequent colonoscopies compared to less frequent. In sum, our prospective observational data confirm neither lower incidence nor lower stage of CRCs at diagnosis when shorter intervals between colonoscopies are recommended.

It has been argued that the effectiveness of a 3-yearly interval in Finland is influenced by a less severe phenotype associated with the founder *path_MLH1* variant. We analyzed the non-Finnish data separately and found a similar lack of association, indicating that our findings were not explained by the Finnish series having different pathogenic variants to the others. We acknowledge that the aim of the present study was not to study the effects of different classes of *path_MMR* variants which would need different methods and which we aim to do later.

We have chosen to use a statistically simple method comparing the different intervals between colonoscopies applied in different centres, as well as longer intervals. This way of categorizing data and results is of interest when calculating cost-efficiency of the different strategies for healthcare.

We lack the stage and/or interval information on about 70% of the total patients registered in the PLSD at the moment. However, we do have complete information from the centres contributing to this study (Table 1). All centres having contributed to the previous PLSD reports were offered the possibility to contribute.

We do not have detailed information on caecal intubation rate, success of bowel preparation or previous adenoma detection rate that are acknowledged key performance indicators associated with colonoscopy quality in sporadic CRC prevention [24]. Although there is limited evidence that they may be associated with the risk of CRC in LS [25], we have no reason to assume that our observation that stage distribution is similar across the different surveillance intervals, is biased by the lack of these data.

The results herein support the hypothesis that in LS, more frequent colonoscopies detect cancers that might not have progressed to clinical recognition and clinical significance. Over-diagnosis is well-recognized in screening science especially in relation to prostate and breast cancers [26–29]. Whether these over-diagnosed cancers would have been controlled or even destroyed, through immune and/or other mechanisms or dwell indolently without causing significant morbidity or mortality, has nor been fully clarified.

The relatively good prognosis of CRCs noted in our previous reports from PLSD may reflect that the most but not all cancers produced by the adenoma-carcinoma mechanism are prevented, and the majority of the incident CRCs that we see have a better prognosis because they arise through different carcinogenetic mechanisms. If so, colonoscopy to prevent CRC may have had the expected effect, while (some of) the CRCs that are detected represent different biological tumor phenotypes.

In parallel with the epidemiological studies mentioned above, it is now agreed that *path_MMR* carriers have multiple MMR deficient/MSI cells in macroscopically normal looking crypts in the colorectal epithelium, and that CRC may develop directly from these without a macroscopically visible adenoma precursor [30, 31]. There is a developing understanding that MSI cells present neoantigens on their surfaces that are detectable by the host immune system, making them targets for destruction by the host [32]. Whether or not one of the MMR deficient crypts develops into a CRC may be dependent upon the mutator phenotype rendering a progenitor cell capable of evading the host immune system [33].

The current paper does not aim to discuss these immunological and commonly agreed mechanisms in detail. They are presented and discussed elsewhere, but the topic is offered as supporting evidence that MSI cells may be killed by the host immune system. The probability that a *path_MMR* carrier develops CRC may be considered a balance between the probability of developing precancerous cells and the probability that the immune system will kill them before they become invasive cancers.

Our offered hypothesis is based on spontaneous regression of cancers. The case report by Karakuchi et al. of spontaneous regression of an MSI high cancer in the transverse colon, assumed to be caused by non-germline acquired somatic *MMR* mutations but not tested to exclude germline pathogenic variants, is supportive [34]. This does not exclude other mechanisms, as mentioned above.

Clearly, carcinogenesis is a multifactorial process and other biological explanations may be offered. If our interpretation of the findings we describe above contains some truth, it does not imply that other hypotheses – including the accelerated adenoma-carcinoma sequence, the possibly important role of missed lesions during suboptimal surveillance colonoscopy, and cancers developing without a detectable and therefore intervention-susceptible precursor lesion – are not true. We cannot completely exclude an important influence of variable quality of endoscopy or some other geographical variable in response to surveillance but the simplest and most persuasive explanation is that having progressed quickly, often from an unrecognizable precursor, a proportion of these cancers regress spontaneously and those that survive tend to remain as a localized lesion, unlikely to metastasize early.

## Conclusions

The PLSD was designed to examine whether or not the expectations derived from current paradigms alone are met, with the basic understanding that if current paradigms may not completely explain what we find, there may be additional instrumental factors to consider. The combined epidemiological results from the PLSD now raise the possibility that some LS-associated CRCs may spontaneously regress. We can find no published epidemiological evidence on the effects of surveillance colonoscopy, which are in conflict with our hypothesis. The concept of over-diagnosis is well established in other fields of cancer screening. The growing body of knowledge on how the host immune system fights abnormal cells in *path_MMR* carriers may be interpreted as support for our hypothesis. We suggest that the hypothesis presented here should be further tested because of its scientific interest and especially because the consequences would be of interest to the *path_MMR* carriers and to the providers of health care. A controlled study comparing the longest safe colonoscopy interval to the shortest possible interval should be able to elucidate the existence of possible over-diagnosis caused by intense monitoring.

## Abbreviations

AJCC: American Joint Committee on Cancer
CRC: Colorectal cancer
HNPCC: Hereditary nonpolyposis colorectal cancer
LS: Lynch Syndrome
MMR: Mismatch repair
*path_MLH1*: Pathogenic (disease-causing) variant of the MLH1 gene
*path_MSH2*: Pathogenic (disease-causing) variant of the MSH2 gene
*path_MSH6*: Pathogenic (disease-causing) variant of the MSH6 gene
*path_PMS2*: Pathogenic (disease-causing) variant of the PMS2 gene
